# Special Issue “Sleep Disorders: From Clinical Research to Daily Practice”

**DOI:** 10.3390/jcm12165271

**Published:** 2023-08-13

**Authors:** Francesco Fisicaro, Giuseppe Lanza, Michela Figorilli

**Affiliations:** 1 Department of Biomedical and Biotechnological Sciences, University of Catania, Via Santa Sofia 97, 95123 Catania, Italy; drfrancescofisicaro@gmail.com; 2 Department of Surgery and Medical-Surgical Specialties, University of Catania, Via Santa Sofia 78, 95123 Catania, Italy; 3 Clinical Neurophysiology Research Unit, Oasi Research Institute—IRCCS, Via Conte Ruggero 73, 94018 Troina, Italy; 4 Neurology Unit, Department of Medical Sciences and Public Health, University of Cagliari and AOU Cagliari, Asse Didattico E., SS 554 Bivio Sestu, Monserrato, 09042 Cagliari, Italy; michelafigorilli@gmail.com; 5 Sleep Disorders Center, Department of Medical Sciences and Public Health, University of Cagliari, Asse Didattico E., SS 554 Bivio Sestu, Monserrato, 09042 Cagliari, Italy

Healthy sleep is indissolubly linked to both physical and mental health, as pointed out by evidence showing the negative impact of poor sleep on neurological, psychiatric, cardiovascular, respiratory, metabolic, and immune systems, among others [1,2]. Both poor sleep quality and quantity are also strongly associated with a variety of neurocognitive and psychosocial alterations [3]. Nevertheless, the phenomenology underlying sleep disorders is still largely unknown, although, recently, significant progress in the understanding of some sleep disorders has been achieved, such as in the case of restless legs syndrome (RLS) and REM sleep behavior disorder (RBD), especially regarding their electrophysiological basis and the possibility to non-invasively neuromodulate them [4]. Regarding specific treatment, however, the management of sleep disorders remains a challenge for both clinicians and researchers. Recently, the conventional pharmacological approaches to sleep disorders have been implemented by remarkable advances in the understanding of sleep pathophysiology, which appears to be strongly linked to their neurochemistry, neuroendocrinology, genetics, and immunology. Nevertheless, pharmacological treatment is not always satisfactory, especially for patients with chronic insomnia (CI) or obstructive sleep apnea syndrome (OSAS) who do not adequately respond, or respond with a poor compliance, to continuous positive airway pressure ventilation [5]. In this scenario, the understanding of the complex processes underlying sleep, its homeostasis, and its plasticity may also lead to innovative targeted and evidence-based therapies, based both on novel drugs and non-pharmacological techniques [6].

Nowadays, among the emerging approaches to sleep medicine, non-invasive brain stimulation is a “cutting edge” topic and, among the available techniques, repetitive transcranial magnetic stimulation (rTMS) has been probed as a therapeutic option for some common and disabling sleep disorders, such as CI, OSAS, and RLS, as recently reviewed [7]. The common goal is to improve impaired cerebral functioning or dysfunctional networks by modulating synaptic plasticity and functional connectivity, with a focus on the optimization of targets, stimulation protocols, candidate symptoms, and outcome measures [8]. As an example, gaining novel insights into the complex interactions between central and peripheral neural circuits in the generation of symptoms of RLS is mandatory for a better diagnostic refinement and new therapeutic options [9]. Further insights are provided by the effectiveness of other non-pharmacological tools, such transcranial direct current stimulation (tDCS) and transcutaneous direct spinal stimulation, thereby extending the therapeutic arsenal for this disorder. Regarding RBD, new methods that specifically explore and possibly measure other alterations beyond the brainstem are relevant to determine whether a prodromic neurodegenerative disorder (i.e., Parkinson’s disease and other alpha-synucleinopathies) underlies this condition occurring even at the stage of isolated RBD [10,11].

With this in mind, in this Special Issue, we focused on both neurophysiological and clinical features of sleep disorders in order to provide new hints towards their pathophysiological mechanisms and, translationally, to guide new treatments. Herein, an overview of five original high-quality scientific contributions included in the above-titled Special Issue collection is provided.

Based on previous findings in patients with isolated RBD [12], Lanza and colleagues [13] used TMS to assess the neurophysiological features in isolated RBD compared to early Parkinson’s disease with RBD. The resting motor threshold, contralateral cortical silent period, amplitude and latency of the motor-evoked potentials, short-interval intracortical inhibition, and intracortical facilitation (ICF) were recorded from 15 drug-naïve iRBD patients, 15 drug-naïve PD with RBD patients, and 15 healthy participants from the right first dorsal interosseous muscle. The REM sleep atonia index, Mini Mental State Examination, Geriatric Depression Scale, and Epworth Sleepiness Scale (ESS) were assessed. The authors found that iRBD and PD with RBD patients shared a reduced ICF, thus suggesting the involvement of glutamatergic transmission both in subjects at risk for degeneration and in those with an overt alpha-synucleinopathy [13]. These results, although promising, prompt follow-up examinations in order to confirm whether the TMS changes observed at this stage will correlate with clinical progression and response to treatment, as well as support the differential diagnosis between different parkinsonian syndromes [14].

Sleep disturbances are common among athletes, with a higher prevalence compared to non-athletic subjects [15,16]. Given the growing interest in the use of neuromodulation techniques for the treatment of sleep disorders, a new research protocol proposed by Etoom and coworkers to assess the efficacy of tDCS on sleep in athletes was particularly timely [17]. To this end, a randomized controlled trial will be performed to test the hypothesis that anodal (excitatory) tDCS over the dorsolateral prefrontal cortex, bilaterally, would improve sleep in different sports athletes. Namely, 84 athletes will be selected based on specific eligibility criteria (i.e., ≥16 years age and global score > 5 on the Pittsburgh Sleep Quality Index (PSQI)) and randomly allocated to the intervention or control group. Each participant will receive a 20 min session of bilateral anodal tDCS with an intensity of 1.5 mA (0.057 mA/cm^2^) in density, three times a week for two weeks. The tDCS will be delivered only for 30 s in the control group. The study’s outcomes are a set of both subjective and objective sleep parameters, which include sleep monitoring, the Insomnia Severity Index (ISI), the ESS, the PSQI, the Depression Anxiety Stress Scale-21, and the Short-Form Survey 12-Item. The results of this ongoing study will hopefully provide an advancement in and contribution to sleep management among athletes.

There is a growing interest in the clinical significance of nocturnal blood pressure (BP), as low quality of sleep and any sleep disturbances may lead to significant increases in nocturnal BP values. In this context, there is increasing evidence that periodic limb movements in sleep (PLMSs) may lead to increased BP values during the night. The featured paper by Krzyzaniak and colleagues suggests a relationship between the number of PLMS and the values of nocturnal BP [18]. To this end, 100 polysomnographic (PSG) recordings of patients with disordered sleep were retrospectively analyzed, and the authors compared the BP of patients with an increased number of PLMSs during the night to the BP of patients with a PLMS number within the normal range. Patients with an increased number of PLMSs had significantly higher values of both systolic BP during the night (119.7 mmHg vs. 113.3 mmHg), sleep (119.0 mmHg vs. 113.3 mmHg), and wake (122.5 mmHg vs. 117.2 mmHg) periods and diastolic BP during the night (75.5 mmHg vs. 70.6 mmHg) and wake (77.6 mmHg vs. 71.5 mmHg) periods compared to controls. These results suggest a relationship between the number of PLMSs during the night and the values of nocturnal blood pressure. Further investigation will clarify if treatment of the sleep disorder might lower nocturnal BP in patients with sleep disorders, therefore improving their vascular risk profile.

As is widely known, the COVID-19 pandemic has just been overcome, although its role on sleep disorders has been widely recognized, and its effects might be present even long after the pandemic ends. Insomnia is prevalent among the general population, and studies have indeed shown an increase in insomnia symptoms during the pandemic. In this context, the network analysis on 785 non-infected subjects by Cha and collaborators aimed to disclose the role of dysfunctional cognitions in the etiological models of insomnia. The ISI, Stress and Anxiety to Viral Epidemics—6 Scale (SAVE-6), and Patient Health Questionnaire—9 (PHQ-9) were used to measure insomnia symptoms, anxiety response to COVID-19, and depression, respectively. Briefly, the network revealed that worrying about current sleep pattern (ISI7) to be the most central insomnia symptom. ISI7 is a measure of the cognitive aspect of insomnia, measuring how severely one worries about their sleep problems rather than the sleep problems themselves. ISI7 was strongly connected to SAVE-6 total score, and ISI2 (i.e., difficulty staying asleep) was strongly connected to the PHQ-9 total score. Overall, this finding supports the role of dysfunctional cognition in the etiological model of insomnia, thus providing the rationale for cognitive behavioral therapy for this condition. The relationship between ISI7 and SAVE-6 might be explained by the transposition of worry and fear of contracting COVID-19 to worry about sleep patterns. Furthermore, the link between ISI2 and PHQ-9 encourages further investigations on whether specific symptoms of insomnia may more frequently associated with depression [19].

Finally, a prospective, longitudinal study investigating sleep-disordered breathing (SDB) in acute stroke patients by Plomaritis and coworkers showed the importance of PSG implementation in everyday clinical practice for acute stroke work-up and management [20]. SDB is common among acute stroke patients; therefore, the authors aimed to investigate the prevalence, severity, and type of SDB in consecutive patients. In more detail, a total of 130 consecutive acute stroke patients who underwent overnight PSG within 72 h from the symptom onset were prospectively evaluated. The rate of SDB detection on PSG recordings was 79% (95% CI: 71–86). Three variables were independently and significantly associated with the likelihood of SDB detection in multivariable analyses adjusting for potential confounders: age (OR per 10-year increase: 2.318, 95% CI: 1.327–4.391), male sex (OR: 7.901, 95% CI: 2.349–30.855), and abnormal ESS score (OR: 6.064, 95% CI: 1.560–32.283). Among patients with SDB, congestive heart failure was independently and significantly associated with the likelihood of central apnea (OR: 18.295, 95% CI: 4.464–19.105). Additionally, among all patients, increasing National Institutes of Health Stroke Scale score on admission (OR: 0.817, 95% CI: 0.737–0.891) and Apnea–Hypopnea Index (OR: 0.979, 95% CI: 0.962–0.996) emerged as independent and significant predictors of excellent functional outcome at three months (modified Rankin Scale scores 0–1). Globally, the results of this study highlight the importance of PSG implementation in everyday clinical practice as well as in acute stroke settings and management.

To conclude, this Special Issue has produced different relevant papers that will help clinicians in their decision making and treatment choices, also providing interesting hints for future research projects. As Guest Editors, we would like to thank all the authors for their valuable contributions, the reviewers for their time in critically reviewing the papers and posing insightful comments, and the editorial team of the *Journal of Clinical Medicine* for their collective support and constant assistance.
